# Recovery of molybdenum using adsorption/desorption by nickel–aluminum or nickel–aluminum–zirconium complex hydroxides from aqueous media

**DOI:** 10.1039/d5ra02921j

**Published:** 2025-07-14

**Authors:** Fumihiko Ogata, Mizuki Kurazaki, Noriaki Nagai, Yugo Uematsu, Megumu Toda, Masashi Otani, Chalermpong Saenjum, Shigeharu Tanei, Naohito Kawasaki

**Affiliations:** a Faculty of Pharmacy, Kindai University 3-4-1 Kowakae Higashi-Osaka Osaka 577-8502 Japan kawasaki@phar.kindai.ac.jp; b Kansai Catalyst Co., Ltd 1-3-13, Kashiwagi-cho, Sakai-ku Sakai Osaka 590-0837 Japan; c Faculty of Pharmacy, Chiang Mai University Suthep Road, Muang District Chiang Mai 50200 Thailand; d Center of Excellence for Innovation in Analytical Science and Technology for Biodiversity-based Economic and Society (I-ANALY-S-T_B.BES-CMU), Chiang Mai University Chiang Mai 50200 Thailand; e Faculty of Pharmaceutical Sciences, Nihon Pharmaceutical University 10281 Komuro, Ina-machi, Kitaadachi-gun Saitama 362-0806 Japan; f Antiaging Center, Kindai University 3-4-1 Kowakae Higashi-Osaka Osaka 577-8502 Japan

## Abstract

The potential of nickel–aluminum (NA series) and nickel–aluminum–zirconium (NAZ series) complex hydroxides in the recovery of molybdenum (Mo) ions was assessed in this study. Batch adsorption experiments, elemental distributions, binding energy analyses, and desorption experiments were performed. The amount of adsorbed Mo increased with increasing aluminum content (NA41 < NA31 < NA21 < NA11 < NA12). Additionally, the incorporation of zirconium in NA did not significantly affect the Mo ion adsorption capacity. The optimal conditions for Mo adsorption were determined. A pH of 5.0 was the most suitable for Mo ion adsorption. The adsorption equilibrium was reached approximately 3 h after the start of adsorption. The adsorbed amount did not change at adsorption temperatures from 7 to 45 °C. To elucidate the adsorption mechanism, ion exchange capacity, elemental distribution, and binding energy analyses were conducted. The results showed that ion exchange with sulfate ions in the adsorbent interlayer and surface characteristics had a significant impact on the adsorption capacity. Finally, a sodium sulfate solution was used as a desorption agent for the recovery of Mo ions from the aqueous phase. These findings offer important insights into the adsorption and desorption of Mo ions from aqueous environments using the NA or NAZ series adsorbents. The recovery of rare metals from aqueous environments can be greatly aided by the treatment recommended in this study.

## Abstract 2

The potential of nickel–aluminum (NA series) and nickel–aluminum–zirconium (NAZ series) complex hydroxides in the recovery of molybdenum (Mo) ions was assessed in this study.

## Graphical abstract

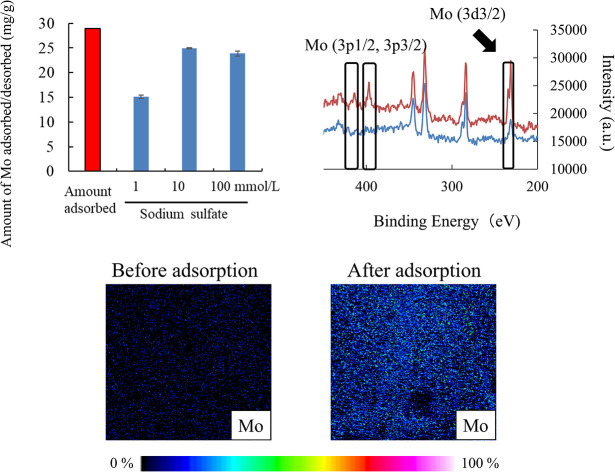

## 1 Introduction

Molybdenum (Mo) is a transition element that is biologically active and necessary for both plants and animals.^1^ However, when its concentration in water is higher than 5 mg L^−1^, it turns hazardous.^2^ Excessive Mo exposure can cause anaemia and thyroid, kidney, and liver dysfunction. Additionally, chronic consumption of a large amount of dietary Mo might be lethal. Therefore, the United States Environmental Protection Agency (U.S. EPA) and the World Health Organization have set the maximum allowable content of Mo in drinking water at 40 μg L^−1^ and 70 μg L^−1^, respectively.^3,4^ Mo pollution of surface water is a significant environmental problem caused by effluents that contain Mo and are released from mining tailings. This phenomenon is evident worldwide, including the United States, Canada, and China.^2,5,6^ Nonetheless, Mo is a rather uncommon metal with a number of possible uses in the metallurgical sector. It is stockpiled in Japan because it is imported; therefore, it is subject to global supply shortages. The demand for Mo has increased in recent years because of its limited deposits in primary sources. Therefore, development of friendly approaches for both removal and recovery of Mo from environmental water is important.^7,8^

Molybdate (MoO_4_^2−^) is the most soluble Mo anion and the most prevalent molybdenum oxyanion.^9,10^ Mo is extracted and recovered from aqueous solutions using numerous techniques, including solvent extraction, ion exchange, adsorption, and chemical precipitation.^11^ When compared to other treatments, adsorption is the most extensively used of them due to its great efficiency, low cost, and simplicity of usage.^12^ Therefore, it is crucial to evaluate inexpensive, environmentally friendly adsorbents with high Mo adsorption capabilities.

Metal complex hydroxides, including nickel–aluminum (NA series) and nickel–aluminum–zirconium complex hydroxides (NAZ series), show excellent removal and recovery capacity for oxyanions of rare and heavy metals (chromium, tungsten, phosphate, and arsenic ions).^13–16^ In particular, the incorporation of zirconium (Zr^4+^) in metal complex hydroxides strongly affects their physicochemical characteristics and positively affects the removal and recovery of oxyanions. Additionally, some adsorption mechanisms between metal complex hydroxides and rare metals in aqueous media have been elucidated.^17^ This information is useful for the application of these metal complex hydroxides in the field. However, no information is available regarding the recovery of Mo by adsorption/desorption using NA and NAZ series. Therefore, if the adsorption and recovery of Mo using these metal complex hydroxides could be explored, the usefulness and applicability of these materials would increase significantly.

Therefore, Mo adsorption and recovery using NA and NAZ series were the primary focus of this investigation. Additionally, we also evaluated the various effects on adsorption performance, including temperature, pH, exposure time, initial concentration, and selectivity.

## 2 Experimental section

### 2.1 Materials

At varying molar ratios, nickel (Ni)–aluminum (Al) or nickel (Ni)–aluminum (Al)–zirconium (Zr) complex hydroxides were produced and their physicochemical characteristics have been reported previously.^15,16^ Briefly, the molar ratios of Ni : Al were 4 : 1, 3 : 1, 2 : 1, 1 : 1, and 1 : 2 which were denoted as NA41, NA31, NA21, NA11, and NA12, respectively. In addition, those of Ni : Al : Zr were 0.9 : 1.0 : 0.09 (NAZ1) and 0.9 : 2.0 : 0.09 (NAZ2). Sodium hydroxide, hydrochloric acid, and standard Mo solutions (Mo in 0.4 mol per L HCl and 0.2 mol per L HNO_3_ concentrations) were provided from FUJIFILM Wako Pure Chemical Co., Ltd (Japan).

### 2.2 Adsorption capacity of prepared adsorbents for Mo

To evaluate the prepared adsorbents' Mo adsorption capability, the following screening experiment was conducted: 0.05 g of each adsorbent and 50 mL of a standard Mo solution with 50 mg L^−1^ were reacted, and the suspension was shaken at 100 rpm for 24 h at 25 °C. Following the reaction, a 0.45 μm membrane filter was used to filter the sample solution. Then, the level of Mo in the obtained solution was measured using an iCAP-7600 Duo; an inductively coupled plasma optical emission spectrometer (Thermo Fisher Scientific Inc., Japan). Finally, the difference between the initial and equilibrium Mo concentrations was used to compute the amount of adsorbed Mo.

### 2.3 Effect of various parameters on the adsorption of Mo

Initially, the impact of pH on Mo adsorption was examined. 0.05 g of NA11 or NAZ1 was mixed with 50 mg L^−1^ of an Mo solution in the volume of 50 mL. With the use of sodium hydroxide or hydrochloric acid, the pH of the reacted solution was adjusted to 3, 5, 7, 9, or 11. Additionally, to evaluate the effect of exposure time during 5 min to 24 h, 0.05 g of NA11 or NAZ1 was mixed with 50 mg L^−1^ of an Mo solution in the volume of 50 mL. Furthermore, to study the effects of the initial concentration and temperature, 0.05 g of NA11 or NAZ1 was reacted with different concentrations, 10 to 50 mg L^−1^, of an Mo solution. The suspension solution was shaken at 100 rpm for 24 h at 7, 25, and 45 °C. Following the reaction, a 0.45 μm membrane filter was used to filter the sample solution. Then, the level of Mo in the obtained solution was measured and the amount of adsorbed Mo was calculated. Finally, to evaluate the adsorption mechanism of Mo, the JXA-8530 (JEOL, Tokyo, Japan) and AXIS-NOVA (Shimadzu Co., Ltd, Kyoto, Japan) were used to examine the electron spectrum and elemental distribution, respectively. The DIONEX ICS-900 instrument (Thermo Fisher Scientific, Inc., Tokyo, Japan) was used to analyze the sulfate ions released from NA11 or NAZ1 which the experimental conditions have been previously reported.^16^

### 2.4 Effect of coexistences on the adsorption of Mo

For assessing the selective adsorption of Mo in the aqueous phase, 0.05 g of NA11 or NAZ1 was added to a complex solution system (50 mg L^−1^ of an Mo solution and 50 mg L^−1^ of nitrate, sulfate, or phosphate ions). Each anion was prepared using sodium nitrate, sodium sulfate, or sodium dihydrogen phosphate. All reagents were purchased from FUJIFILM Wako Pure Chemical Ind., Ltd (Special Grade Reagent, Japan). The complex solution was shaken at 100 rpm for 24 h at 25 °C. Following the reaction, a 0.45 μm membrane filter was used to filter the sample solution and the level of Mo in the obtained solution was measured. The DIONEX ICS-900 instrument was used to measure each anion substant. The 12.5 mmol per L sulfuric acid solution was used as regenerant and 2.7 mmol per L sodium carbonate solution with +0.3 mmol per L sodium hydrogen carbonate solution was used as mobile phases with the flow rate of 1.0 mL min^−1^ and the injection volume was set at 10 μL under ambient conditions.

### 2.5 Recovery of Mo based on adsorption/desorption using NA11 or NAZ1

The adsorption/desorption capacity for Mo was assessed to confirm its recovery from the aqueous phase. NA11 or NAZ1 (0.5 g) was reacted with a 100 mg per L Mo solution (300 mL) prepared using disodium molybdate (vi) dihydrate. The adsorption capacity was calculated using a previously described procedure.^18^ Following adsorption, the adsorbent was collected, dried, and used in desorption experiments. The dried adsorbent was reacted with 50 mL of various concentrations desorption solution, including 1, 10, or 100 mmol L^−1^ of hydrochloric acid, sodium hydroxide, or sodium sulfate solution. The mixture was shaken at 100 rpm for 24 h at 25 °C. Following the desorption, a 0.45 μm membrane filter was used to filter the sample solution and the Mo concentration was measured. Finally, the amount of desorbed Mo was calculated as the difference between the initial and equilibrium concentrations. This adsorption and desorption cycle constituted one iteration, and this recycling experiment was conducted thrice.

## 3 Results and discussion

### 3.1 Characteristics of prepared adsorbents

The physicochemical characteristics of NA and NAZ adsorbents have previously reported.^14,19^ Briefly, the NA and NAZ adsorbents were not perfectly spherical and no significant differences were observed between the adsorbents. An amorphous structure was observed with increasing amounts of Al in the NA adsorbent series. Moreover, the XRD patterns of NAZ1 and NAZ2 showed trends similar to those of NA11 and NA12, respectively. These phenomena suggest that the constituent metals, such as Ni or Al, significantly affect the crystal system. Moreover, in the prepared NA or NAZ adsorbents, these parameters have a significant impact on the distance between the constituent metals as well as the distance between the interlayer region and the brucite-like layer. The specific surface areas of NA41, NA31, NA21, NA11, NA12, NAZ1, and NAZ2 were 14.6, 11.7, 15.6, 22.8, 26.4, 51.9, and 27.8 m^2^ g^−1^, respectively. The amount of hydroxyl groups in NA41, NA31, NA21, NA11, NA12, NAZ1, and NAZ2 were 0.80, 0.82, 1.05, 1.92, 1.62, 1.08, and 1.51 mmol g^−1^, respectively. The pH_pzc_ of the adsorbents ranged from 6.0 to 6.4 in this study.

### 3.2 Amount of Mo adsorbed using NA and NAZ adsorbents

**Fig. 1:**
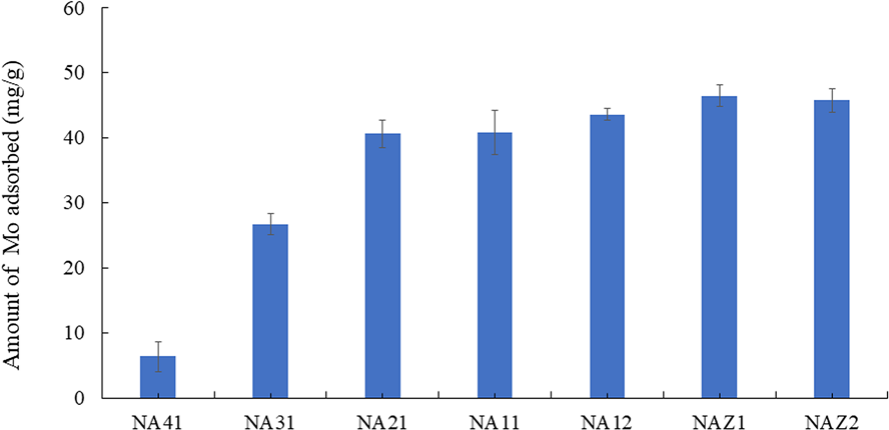
Adsorption capacity of Mo onto adsorbents. Initial concentration: 50 mg L^−1^, sample volume: 50 mL, adsorbent: 0.05 g, temperature: 25 °C, contact time: 24 h, 100 rpm.

The adsorption capacities for Mo using NA41, NA31, NA21, NA11, NA12, NAZ1, and NAZ2 were 6.3, 26.7, 40.3, 40.9, 43.6, 46.5, and 45.8 mg g^−1^, respectively (Fig. 1). Under our experimental conditions, in the NA adsorbent series, the adsorbed amount increased with increasing amounts of Al. Additionally, no significant differences were observed between the NA and NAZ adsorbents. The addition of Zr to the NA adsorbents did not significantly increase the adsorption capacity for Mo from the aqueous phase. The adsorption capacity for Mo may be correlated with the specific surface area and quantity of hydroxyl groups. However, to clarify the correlation between the adsorption capacity and the adsorbent's physicochemical properties, further thorough research is necessary. In this study, NA11, NA12, NAZ1, and NAZ2 (mainly NA11 and NAZ1), which have high adsorption capabilities for Mo, were used in the subsequent experiments.

### 3.3 Factors affecting the adsorption of Mo

One crucial regulatory factor for Mo adsorption is pH. Previous studies have reported the relationship between predominance diagram electric potential (*E*_h_) and pH. In the central region of *E*_h_–pH diagram, the predominant species is MoO_2_, whilst for reducing potential metallic Mo predominates.^20,21^ In the presence of diluted acid such as HCl and/or H_2_SO_4_, Mo(vi) reduction can occur giving some soluble complexes.^20^ (a) In acid solutions, the passive film consists mainly of MoO_2_ together with MoO_3_ and Mo(OH)_3_. (b) In neutral solutions, the reduction reaction may occur by formation of MoO_2_. (c) In basic solution, MoO_3_ is reduced according to (b).^22^

**Fig. 2:**
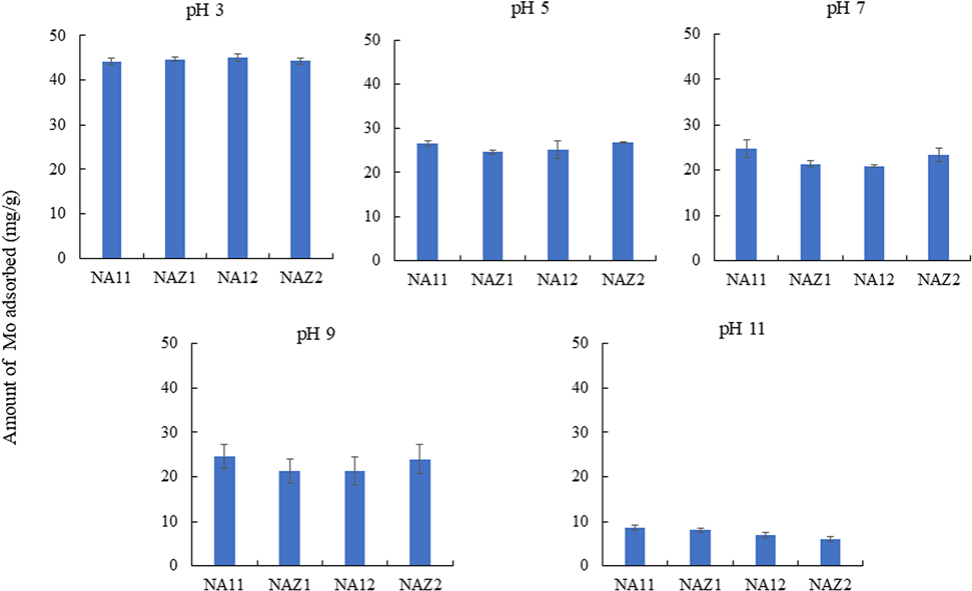
Adsorption capacity of Mo at different pH conditions. Initial concentration: 50 mg L^−1^, sample volume: 50 mL, adsorbent: 0.05 g, temperature: 25 °C, contact time: 24 h, 100 rpm.

In this study, the molybdate speciation occurs as follows: H_2_MoO_4_ predominates in the solution at a pH of 2. HMoO_4_^−^ begins to form at a pH of approximately 1.75 and reaches a concentration of 30% at a pH of 4.0. MoO_2_^2−^ is first formed at a pH of approximately 3.0. MoO_2_^2−^ is the dominant species in the pH range of 5.0–14.0. H_2_MoO_4_ can protonate (H_3_MoO_4_^+^), but only in strongly acidic solutions, and plays no role in adsorption. All three speciations (H_2_MoO_4_, HMoO_4_^−^, and MoO_2_^2−^) can be found in solutions in the pH range of 3–5.^9^ The amount of adsorbed Mo decreases with increasing pH (pH 2 > pH 11). The adsorbent surface was positively charged (the value of pH_pzc_ was approximately 6.2–6.4), and the reaction between Mo and the adsorbent easily occurred under acidic pH conditions (Fig. 2). However, under basic conditions, repulsion occurred easily between Mo and hydroxide ions or the negatively charged adsorbent surface. Therefore, the Mo adsorption capacity was decreased significantly in this study. In addition, the concentrations of constituent metals released from the adsorbents were measured (data not shown). Ni and Al were detected at a pH of 3, whereas Al was detected at a pH of 11. These phenomena indicate that the prepared adsorbent was partially destroyed at the pH 3 and 11. Therefore, a pH of 5 was selected for Mo adsorption in subsequent experiments. Similar patterns have been noted in a previous study.^2^

**Fig. 3:**
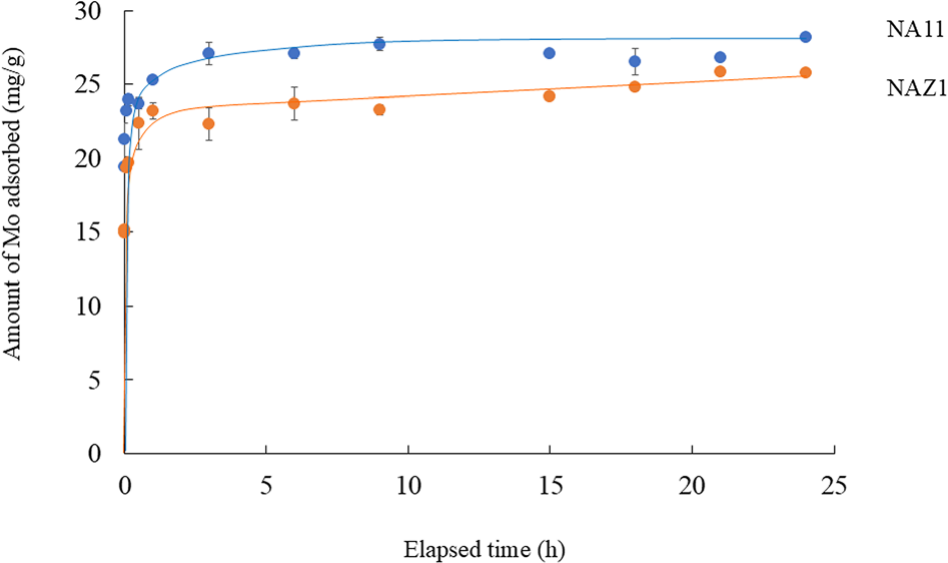
Effect of contact time on the adsorption of Mo. Initial concentration: 50 mg L^−1^, sample volume: 50 mL, adsorbent: 0.05 g, temperature: 25 °C, contact time: 5 min–24 h, 100 rpm.

Fig. 3 demonstrated the effect of contact time on the adsorption of Mo using the NA and NAZ. Adsorption rapidly increased at 0.5 h, and reached equilibrium within 3 h. In this study, both common kinetic models, namely the pseudo-first-order model (PFOM) and pseudo-second-order model (PSOM), were utilize to evaluate the adsorption of Mo.^23,24^ PFOM was used when the number of unoccupied sites on the adsorbent was equal to the occupancy rate of the binding sites. In contrast, PSOM was used when the occupancy rate of the adsorption sites was proportional to the square of the number of unoccupied sites on the adsorbent.^25^Table 1 lists the relevant parameters and the two kinetic models with their correlation coefficients. As shown in Table 1, the correlation coefficient of PSOM was higher than that of PFOM, and the calculated *q*_e,exp_ values were closer to the theoretical *q*_e_,_cal_ values obtained using PSOM. This information indicates that PSOM is the best representation for the adsorption of Mo on NA11 and NAZ1, which shows that the rate-limiting step may be chemical adsorption by sharing or exchanging electrons between the adsorbate and adsorbent.^26^

**Table 1:** Comparison of the two models for Mo adsorption onto NA11 and NAZ1 adsorbents

Sample	*q*_e_,_exp_ (mg g^−1^)	Pseudo-first-order model			Pseudo-second-order model		
		*K*_1_ (h^−1^)	*q*_e,cal_ (mg g^−1^)	*r*	*K*_2_ (mg g^−1^ h^−1^)	*q*_e,cal_ (mg g^−1^)	*r*
NA11	28.2	0.037	3.6	0.624	0.7	27.4	0.999
NAZ1	25.8	0.15	6.7	0.890	0.2	25.5	0.999

**Fig. 4:**
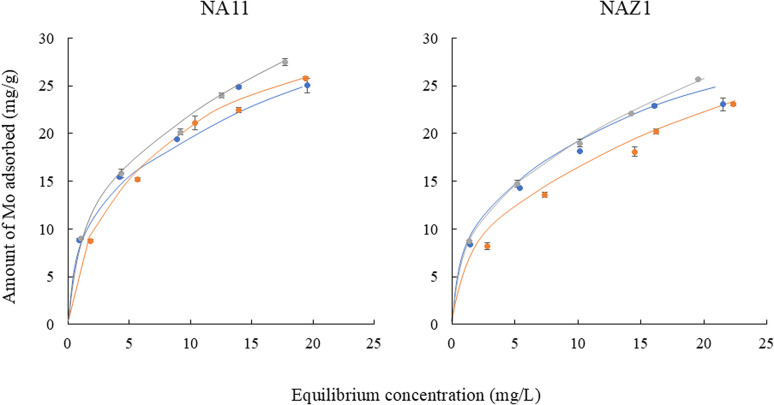
Adsorption isotherms of Mo at different temperatures. Initial concentration 10–50 mg L^−1^, solvent volume 50 mL, adsorbent 0.05 g, pH 5, contact time 24 h, temperature 7(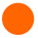), 25(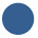), and 45(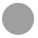) °C, agitation speed 100 rpm.

Fig. 4 displays the Mo adsorption isotherms at different temperatures. The adsorption capacity for Mo did not change significantly between 7 and 45 °C. These data indicate that NA11 and NAZ1 can be used at various temperatures in the field. According to a previous study, adsorption isotherms exhibited a particular correlation between the amount of absorbate and the adsorbate concentration at a constant temperature. Two common isotherm models, namely Langmuir and Freundlich, were fitted to evaluate the performance of Mo.^27,28^

By contrast, the Freundlich isotherm model can be applied to extremely heterogeneous surfaces, and an adsorption isotherm without a saturation plateau may be inactive due to a multilayer adsorption mechanism.^28^

**Fig. 5:**
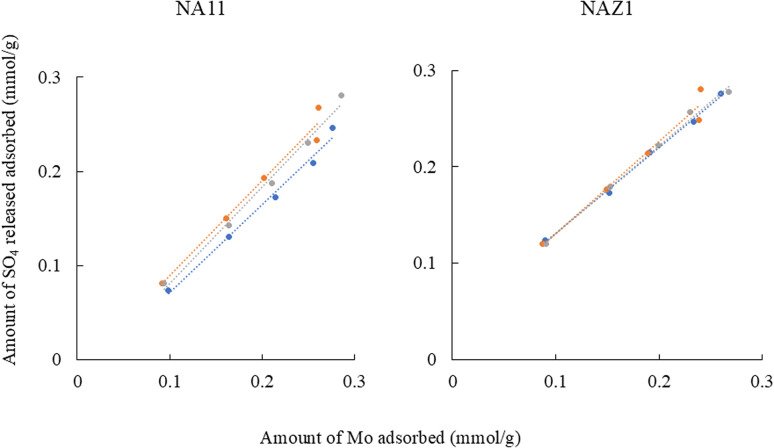
Relationship between amount of Mo adsorbed and amount of sulfate ion released. Initial concentration 10–50 mg L^−1^, solvent volume 50 mL, adsorbent 0.05 g, pH 5, contact time 24 h, temperature 7(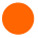), 25(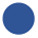), and 45(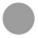) °C, agitation speed 100 rpm.

Fitting results of isotherm data using Freundlich and Langmuir models are displayed in Table 2. The Freundlich model exhibited the correlation coefficient in the range of 0.995–0.999 which is more appropriate than the Langmuir model which demonstrated the correlation coefficient in the range of 0.984–0.995 for describing the adsorption of Mo. The maximum adsorption capacity for Mo (*q*_mas_) using NA11 or NAZ1 did not change significantly between 7 °C and 45 °C. This phenomenon supported the data in adsorption isotherms (Fig. 4). Finally, Mo was easily adsorbed on the NA11 and NAZ1 surfaces when 1/*n* was in the 0.1–0.5 range.^29^ Conversely, when 1/*n* was >2, Mo did not easily adsorb onto the NA11 or NAZ1 surface. The 1/*n* value in this investigation ranged from 0.36 to 0.50, suggesting that Mo was easily adsorbed on NA11 and NAZ1 in our experimental setup. Many researchers have reported the adsorption mechanism of oxyanions using metal complex hydroxides. In particular, electrostatic interaction, ligand exchange, and ion exchange have been evaluated.^18,30–32^ In this study, sulfate ions were maintained as exchangeable anions in the interlayer of NA11 and NAZ1. Fig. 5 demonstrated a positive correlation of 0.987–0.995 for NA11 and 0.985–0.998 for NAZ1 was confirmed, indicating that ion exchanges between the Mo and sulfate ions clearly occurred in this time.

**Table 2:** Fitting results of isotherm data using Freundlich and Langmuir models

Sample	Temperature (°C)	Langmuir isotherm model			Freundlich isotherm model		
		*K*_L_ (L mg^−1^)	*q*_max_ (mg g^−1^)	*r*	Log *K*_F_	1/*n*	*r*
NA11	7	4.9 × 10^−3^	28.6	0.994	0.83	0.46	0.997
	25	2.9 × 10^−3^	24.8	0.984	0.96	0.36	0.995
	45	3.3 × 10^−3^	27.1	0.986	0.94	0.39	0.998
NAZ1	7	7.1 × 10^−3^	24.4	0.995	0.69	0.50	0.998
	25	4.7 × 10^−3^	24.2	0.988	0.87	0.39	0.996
	45	4.5 × 10^−3^	25.6	0.986	0.88	0.41	0.999

**Fig. 6:**
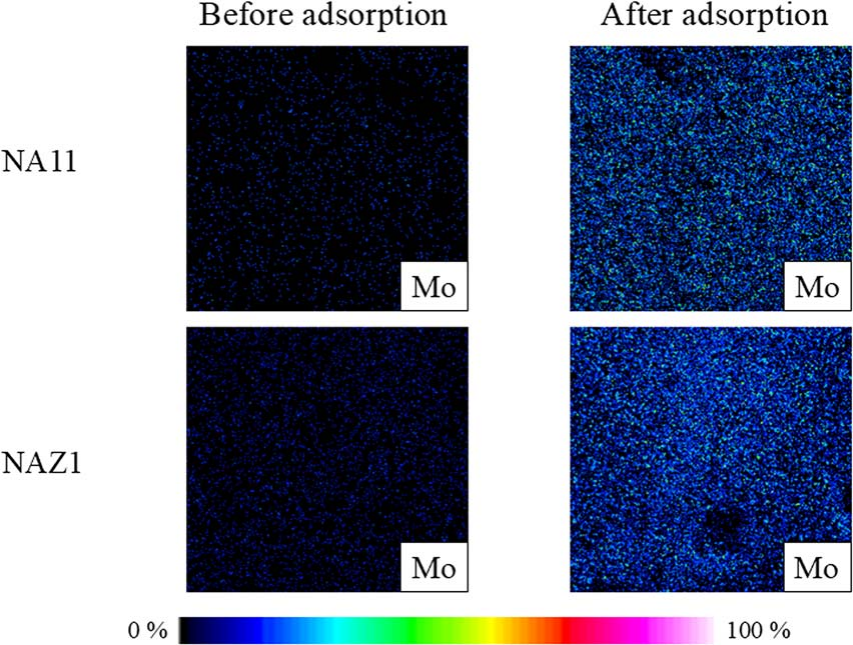
Elemental distributions of Mo before and after adsorption. Initial concentration: 50 mg L^−1^, sample volume: 50 mL, adsorbent: 0.05 g, temperature: 25 °C, contact time: 24 h, 100 rpm.

To further confirm the adsorption of Mo on the NA11 and NAZ1 surfaces. Fig. 6 exerted the elemental distributions of Mo on the adsorbents before and after adsorption which the amount of Mo increased after adsorption. This demonstrates that Mo was adsorbed on the surface of the adsorbent, confirming the previously stated adsorption phenomena.

**Fig. 7:**
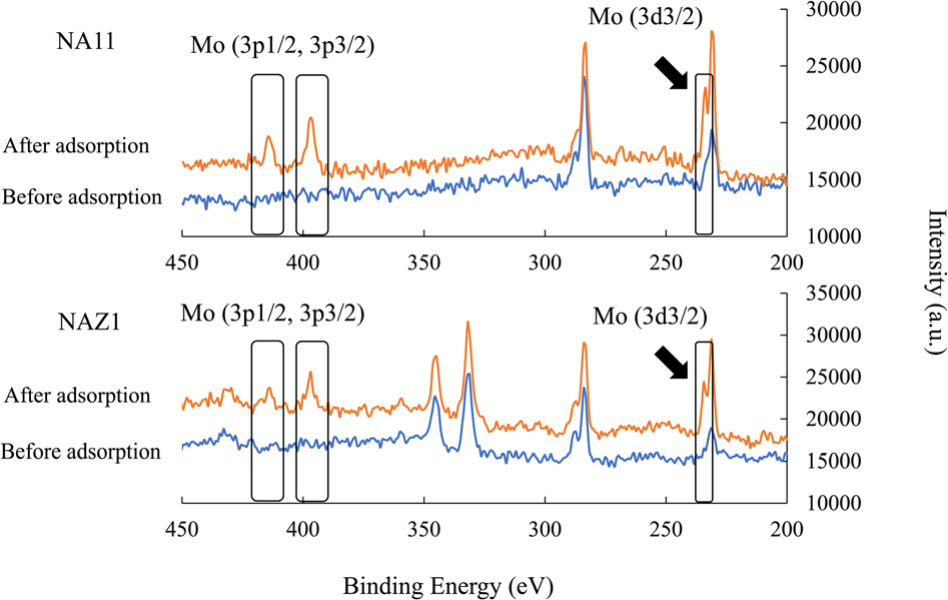
Binding energy of Mo before and after adsorption. Initial concentration: 50 mg L^−1^, sample volume: 50 mL, adsorbent: 0.05 g, temperature: 25 °C, contact time: 24 h, 100 rpm.

Fig. 7 demonstrated the binding energies of Mo that were measured before and after the adsorption. The results exhibited that the intensity of Mo increased after adsorption. These results support the aforementioned ion exchange mechanism. After adsorption, the Mo 3p_1/2_, 3p_3/2_, and 3d_3/2_ XPS peaks, which were not detected before adsorption, became distinct.

**Fig. 8:**
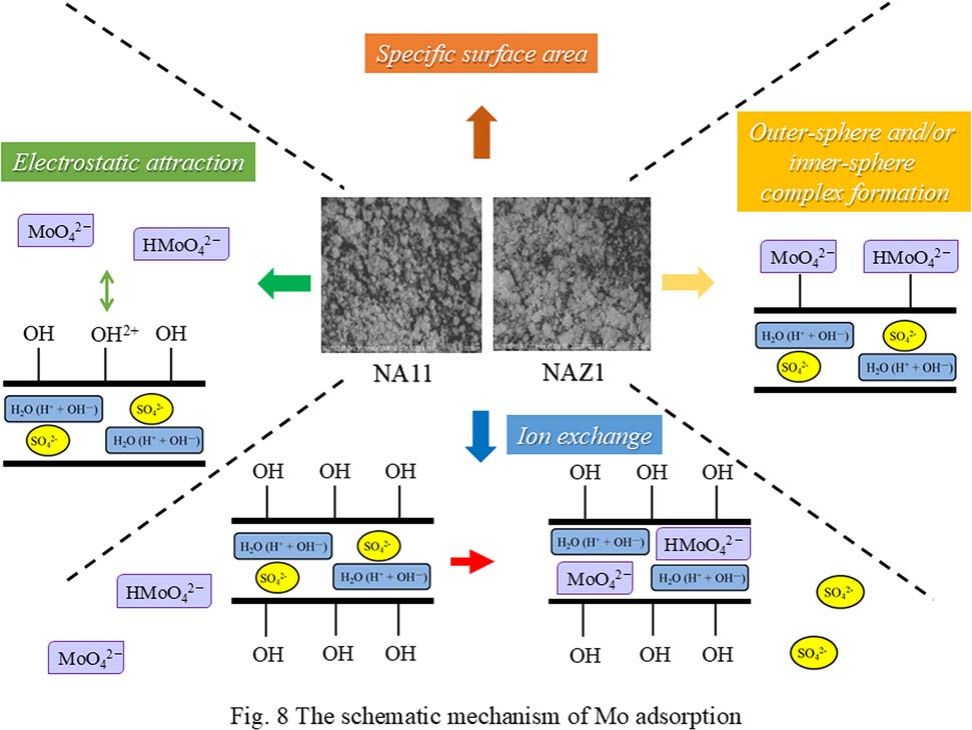
The schematic mechanism of Mo adsorption.

Collectivity, the schematic mechanism of Mo adsorption is shown in Fig. 8. The adsorption mechanisms can mainly be divided into four stages. First, Mo is adsorbed onto the NA11 and/or NAZ1 surface sue to the specific surface area. Second, ion exchange occurs between Mo and sulfate ions in the interlayer of the adsorbents. Third, electrostatic attraction occurs between surface hydroxyl groups and Mo. Finally, Mo such as oxyanion from (Lweis base) directly bonds to the base metals, such as Ni, AL, and Zr (Lweis acid), indicating inner-sphere and/or outer-sphere complex.

### 3.4. Effect of coexistences on the adsorption of Mo

The liquid-phase coexistence of anions may have an impact on Mo's adsorption on NA11 and NAZ1. Therefore, coexistence's impact on the adsorbents' ability to absorb Mo was evaluated (Table 3). In complex solution systems, the amount of Mo adsorbed on NA11 and NAZ1 was 22.9 and 14.9 mg g^−1^, respectively. Nitrate ions were not adsorbed on either of the adsorbents. Conversely, phosphate ions were significantly adsorbed at approximately a concentration of 46.7 mg g^−1^ for NA11 and 47.7 mg g^−1^ for NAZ1. Therefore, phosphate ions slightly or clearly affected the adsorption capacity for Mo using NA11 and NAZ1. Finally, the concentration of sulfate ions increased after adsorption. These phenomena suggest that ion exchange occurs between Mo and/or phosphate ions and sulfate ions in the adsorbent interlayer. This mechanism has been described in the previous section. Finally, this study has a limitation such as the effect of coexistences on the adsorption in detail. Therefore, further studies are needed to elucidate the NA11 and NAZ1 potential as Mo adsorbent.

**Table 3:** Adsorption capacity of anions on NA11 and NAZ1 in complex solution system

Sample	Molybdate ion (mg g^−1^)	Nitrate ion (mg g^−1^)	Sulfate ion^a^ (mg g^−1^)	Phosphate ion (mg g^−1^)
NA11	22.9	0	54.9	46.7
NAZ1	14.9	0	49.4	47.7

a Sulfate ion shows the amount released from NA11 or NAZ1.

### 3.5. Adsorption/desorption capacity for Mo using NA11 and NAZ1

**Fig. 9:**
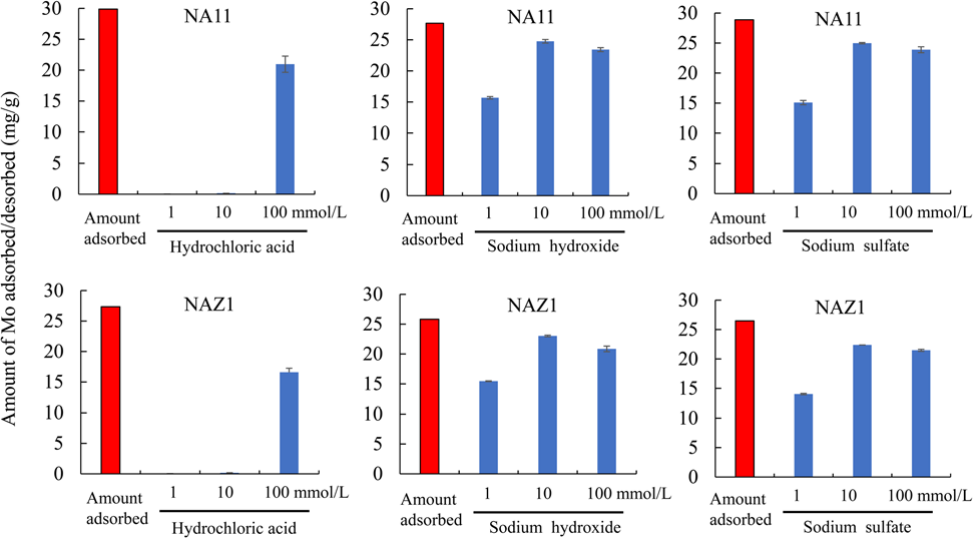
Adsorption/desorption capability of Mo using desorption solution. Adsorption condition (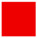): initial concentration: 100 mg L^−1^, sample volume: 300 mL, adsorbent: 0.5 g, temperature: 25 °C, contact time: 24 h, 100 rpm; desorption condition (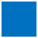): initial concentration: 1, 10, and 100 mmol L^−1^, sample volume: 50 mL, adsorbent: 0.05 g, temperature: 25 °C, contact time: 24 h, 100 rpm.

Fig. 9 shows the adsorption/desorption capacities for Mo using hydrochloric acid, sodium hydroxide, and sodium sulfate solutions at various concentrations. Overall, the amount of desorbed Mo increased with increasing concentrations of the desorption solutions. In particular, hydrochloric acid at a concentration of 1 or 10 mmol L^−1^ was not useful for desorbing Mo from NA11 or NAZ1. In addition, the amount of Mo desorbed using a sodium hydroxide solution showed a trend similar to that using sulfate solutions at 10 or 100 mmol L^−1^ (desorption percentage: 82.7–89.9% for NA11 and 81.0–89.5% for NAZ1). There has been discussion on the optimal pH level for removing Mo from aqueous media, which was 5. Therefore, sodium sulfate solution is useful for the adsorption/desorption of Mo in aqueous media.

**Fig. 10:**
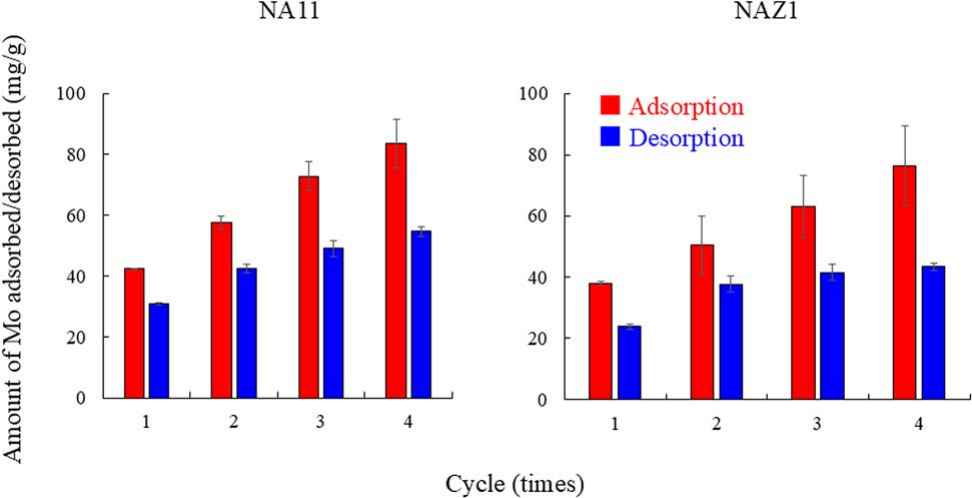
Recycling of NA11 and NAZ1 using sodium sulfate solution. Adsorption condition: initial concentration: 100 mg L^−1^, sample volume: 100 mL, adsorbent: 0.1 g, temperature: 25 °C, contact time: 24 h, 100 rpm, desorption condition: initial concentration: 10 mg L^−1^, sample volume: 100 mL, temperature: 25 °C, contact time: 24 h, 100 rpm, 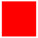 adsorption, 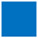 desorption.

**Fig. 11:**
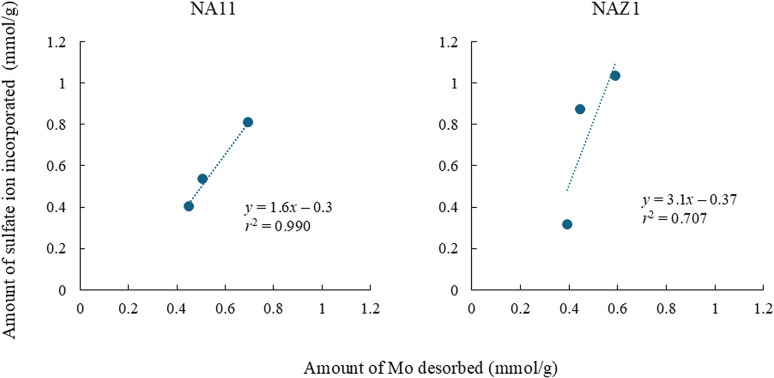
Relationship between amounts of Mo desorbed and amounts of sulfate ion incorporated into adsorbent. Adsorption condition: initial concentration: 100 mg L^−1^, sample volume: 100 mL, adsorbent: 0.1 g, temperature: 25 °C, contact time: 24 h, 100 rpm, desorption condition: initial concentration: 10 mg L^−1^, sample volume: 100 mL, temperature: 25 °C, contact time: 24 h, 100 rpm.

Subsequently, to elucidate the feasibility of NA11 and NAZ1 in recovering Mo, recovery and regeneration experiments were conducted using a 10 mmol per L sodium sulfate solution (Fig. 10). Amount of Mo adsorbed/desorbed increased with increasing cycles. Ion exchange with sulfate ion in the interlayer of prepared adsorbents is one of the adsorption mechanisms of Mo (Section 3.3). In this study, sulfate ions were more incorporated in the interlayer of NA11 and NAZ1 in the desorption process. Therefore, the relationship between amounts of Mo desorbed from adsorbent and amounts of sulfate ions incorporated into the adsorbent was demonstrated (Fig. 11). Correlation coefficient ranged from 0.729 to 0.972 (the slope was 3.1 for NA11 or 4.2 for NAZ1), indicating that sulfate ions were more incorporated into NA11 and NAZ1. These results suggest that the adsorption capacity of Mo using NA11 and NAZ1 increases after the desorption process.

## 4. Conclusions

In this study, Ni–Al (NA series) and Ni–Al–Zr (NAZ series) were prepared, and the adsorption capability for Mo was determined. The effect of optimizing parameters, including temperature (from 7 °C to 45 °C), exposure time (within 3 h), and pH (approximately 5), on Mo adsorption using NA11 or NAZ1 was determined. The adsorption process followed a PSOM (correlation coefficient: 0.999). Mo adsorption is strongly related to ion exchange with sulfate ions in the interlayers of NA11 and NAZ1. The elemental distribution of Mo was evaluated before and after adsorption treatment to determine the adsorption of Mo ions on the adsorbent surface. The intensity of Mo increased following the adsorption process. Finally, sodium sulfate solution was useful for Mo desorption (desorption percentage: 82.7–86.5% for NA11 and 81.1–84.5% for NAZ1). Thus, NA11 and NAZ1 are promising adsorbents for the recovery of Mo from aqueous solutions.

## Author contributions

Fumihiko Ogata: conceptualization, project administration, writing – original draft, and writing – review & editing; Mizuki Kurasaki: investigation and visualization; Noriaki Nagai: investigation and visualization; Yugo Uematsu: investigation and visualization; Megumu Toda: methodology; Masahi Otani: methodology; Chalermpong Saenjum: investigation and visualization; Shigeharu Tanei: methodology; Naohito Kawasaki; project administration, supervision, and writing – review & editing.

## Conflicts of interest

There are no conflicts to declare.

## Data Availability

All data associated with this study have been included in the manuscript.
